# Supplementary material for: TGFβ limits Myc-dependent TCR-induced metabolic reprogramming in CD8+ T cells
Source: Front Immunol. 2022 Jul 26;13:913184. doi: 10.3389/fimmu.2022.913184 (PMC9360539; doi:10.3389/fimmu.2022.913184)
Supplement: Supplementary file 1 [file DataSheet_1.docx]

**TGFβ limits Myc-dependent TCR-induced metabolic reprogramming in CD8^+^ T cells**

Helen Carrasco Hope^1,2^, Gabriella Pickersgill^1^, Pierpaolo Ginefra^2^, Nicola Vannini^2^, Graham P. Cook^1^ and Robert J. Salmond^1^

^1^ Leeds Institute of Medical Research at St James’s, University of Leeds, Wellcome Trust Brenner Building, St James’s University Hospital, Leeds, UK

^2^ Ludwig Institute for Cancer Research, University of Lausanne, Epalinges 1066, Switzerland


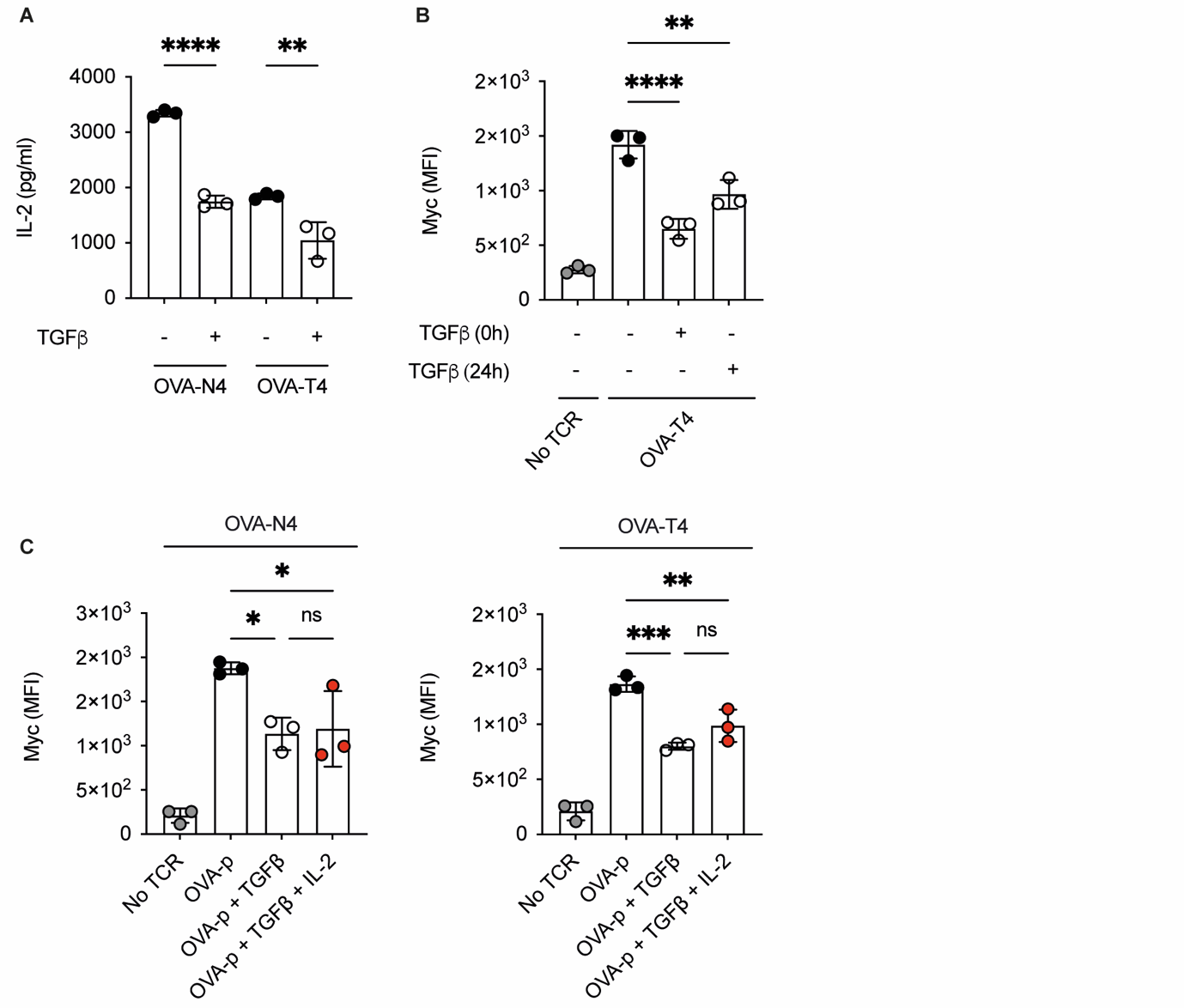


**Supplementary Figure 1. TGFβ-mediated Myc repression is independent of IL-2 depletion.**

OT-I T cells were stimulated with SIINFEKL (OVA-N4) or SIITFEKL (OVA-T4) in the presence or absence of TGFβ for 24h (**A**) or 48h (**B, C**). (**A**) OT-I T cells were stimulated in the presence of CD25 blocking mAb to prevent IL-2 consumption. IL-2 levels in supernatants were determined by ELISA. To assess the role of IL-2 depletion on the TGFβ-mediated repression of Myc expression, OT-I T cells were treated with TGFβ only after 24h of TCR-priming (**B**) or together with hIL-2 (1ng/ml) from timepoint 0h (**B**). Individual data points represent technical replicates from 1 of 3 repeated experiments. ns – not significant, * p<0.05, ** p<0.01, *** p<0.001, **** p<0.0001 as assessed by 1-way ANOVA, with Tukey’s multiple comparisons test.

**Supplementary Table 1. KEGG pathway analysis of upregulated genes by TGFβ.**

List of top 5 pathways upregulated by TGFβ in RNA-Seq dataset of OT-I T cells stimulated with SIITFEKL (OVA-T4) ± TGFβ for 24h, as determined by KEGG analysis using the platform Enrichr. Table includes list of genes identified as differentially expressed (*p* adjust < 0.05, fold-change > 1.5) in RNA-Seq dataset.
